# Supplementary material for: Meiotic sex in Chagas disease parasite Trypanosoma cruzi
Source: Nat Commun. 2019 Sep 3;10:3972. doi: 10.1038/s41467-019-11771-z (PMC6722143; doi:10.1038/s41467-019-11771-z)
Supplement: Supplementary file 1 — Supplementary Information [file 41467_2019_11771_MOESM1_ESM.pdf]

Supplementary Information

Meiotic sex in Chagas disease parasite *Trypanosoma cruzi*

Schwabl *et al.*

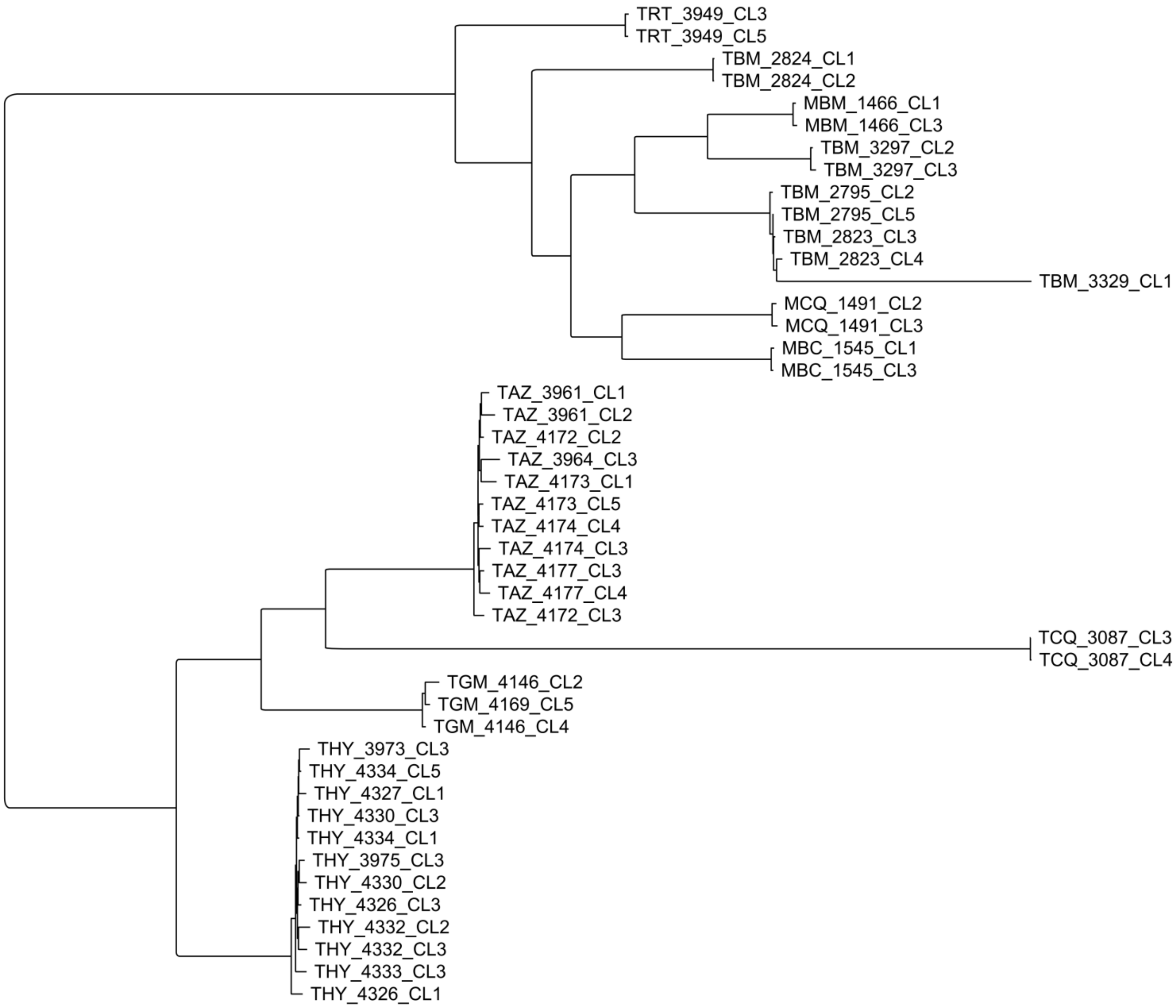

Supplementary Figure 1. Maximum-likelihood phylogenetic relationships among *T. cruzi* I clones**.** Pairwise genetic distances are haplotype-based, defined as the proportion of non-shared alleles across all SNP sites for which genotypes are called in all individuals (*n* = 7,392). The tree follows a general time-reversible (GTR) substitution model with ascertainment bias correction.

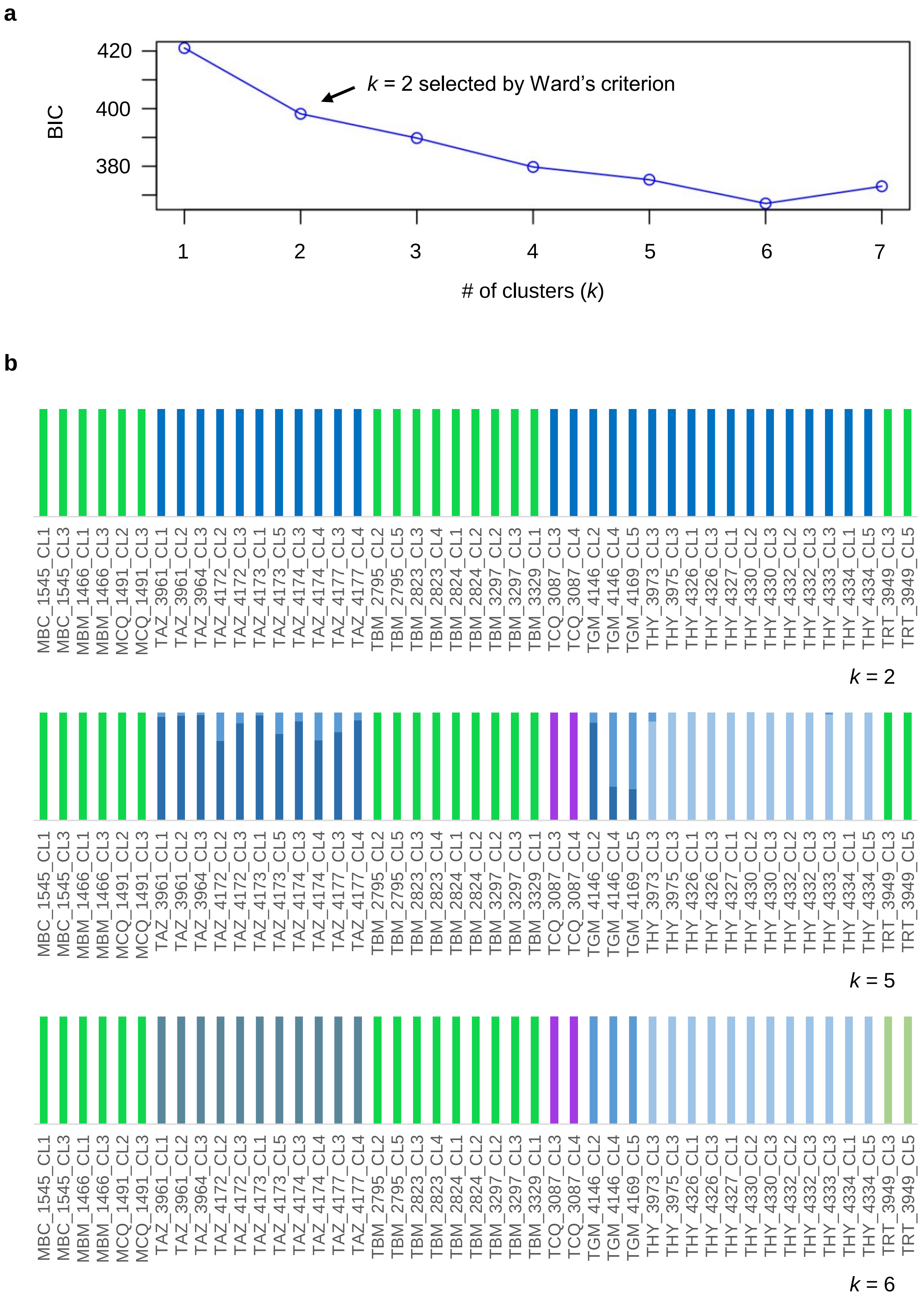

Supplementary Figure 2. Nonparametric population clustering of *T. cruzi* I clones. **a** Bayesian Information Criterion (BIC) scores of k-means clustering solutions for population assignment of *T. cruzi* I clones, based on 68,449 biallelic sites. Ward's criterion[1] was applied for objective selection of *k*. **b** Discriminant analysis of principle components (DAPC) membership probabilities for *k* = 2, *k* = 5 and *k* = 6. Latter k-means solutions allow for additional partitioning of genetic diversity but do not necessarily imply true population subdivision. Colors represent different population assignments.

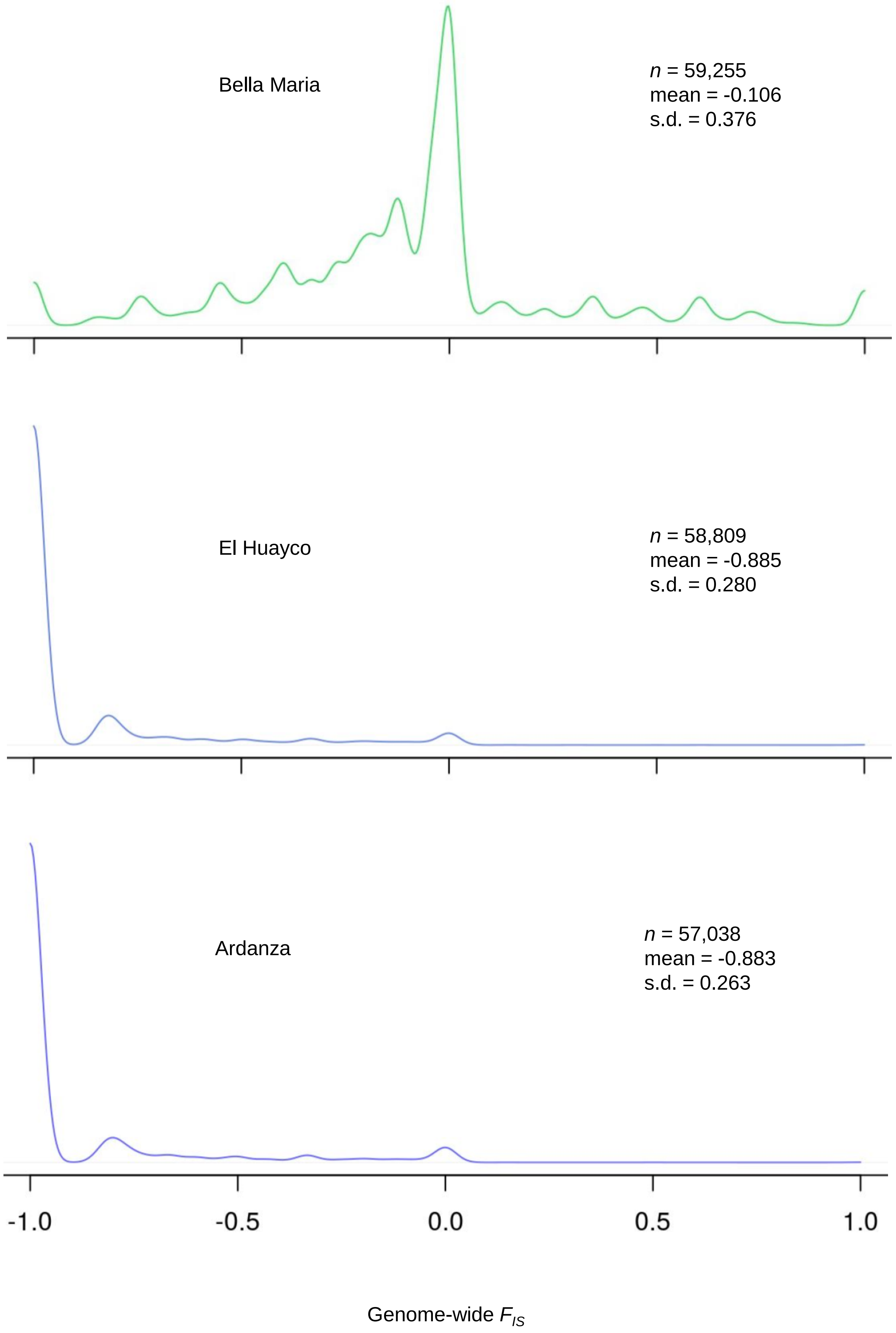

Supplementary Figure 3. Rates of homozygosity relative to Hardy-Weinberg expectations in *T. cruzi* I clones. Genome-wide density distributions of Wright's inbreeding coefficient F_{IS} are plotted for parasites from Bella Maria, El Huayco and Ardanza. F_{IS} sample size, mean and standard deviation are also given for each group, based on the dataset's total 130,996 diagnostic SNPs.

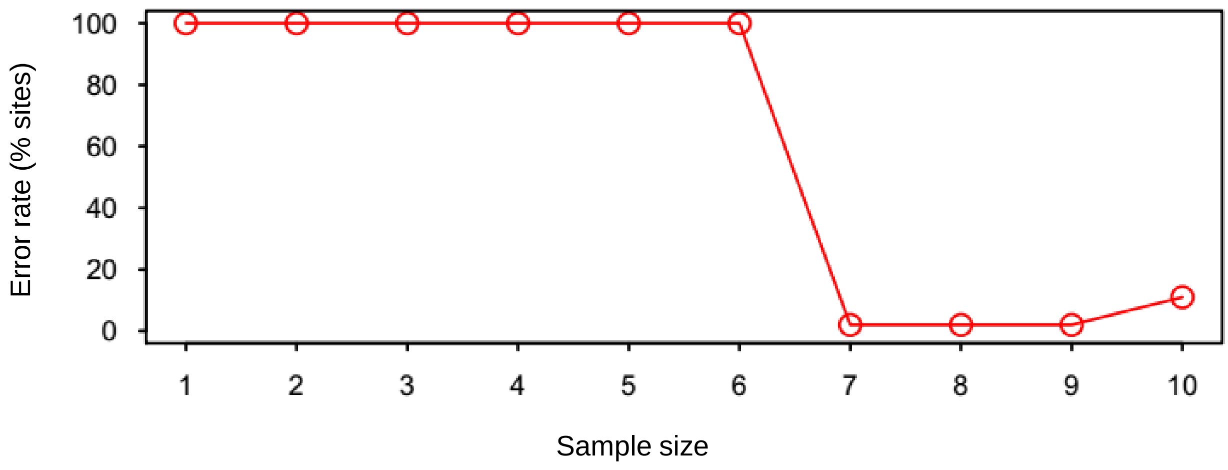

Supplementary Figure 4. Power to reject Hardy-Weinberg equilibrium in asexual genomes. We measured the proportion of SNP sites for which the --hwe function in vcftools[2] incorrectly accepts a null hypothesis of Hardy-Weinberg equilibrium (i.e., $p > 0.05$) in sets of 1 - 10 non-recombinant *T. cruzi* genomes (22,475 SNPs simulated with BamSurgeon[3]; see *Methods*). Type II error predominates when the simulated data is reduced to < 7 individuals, as occurs when observations from Loja are restricted to one clone per vector/host (see Ardanza in Supplementary Table 3).

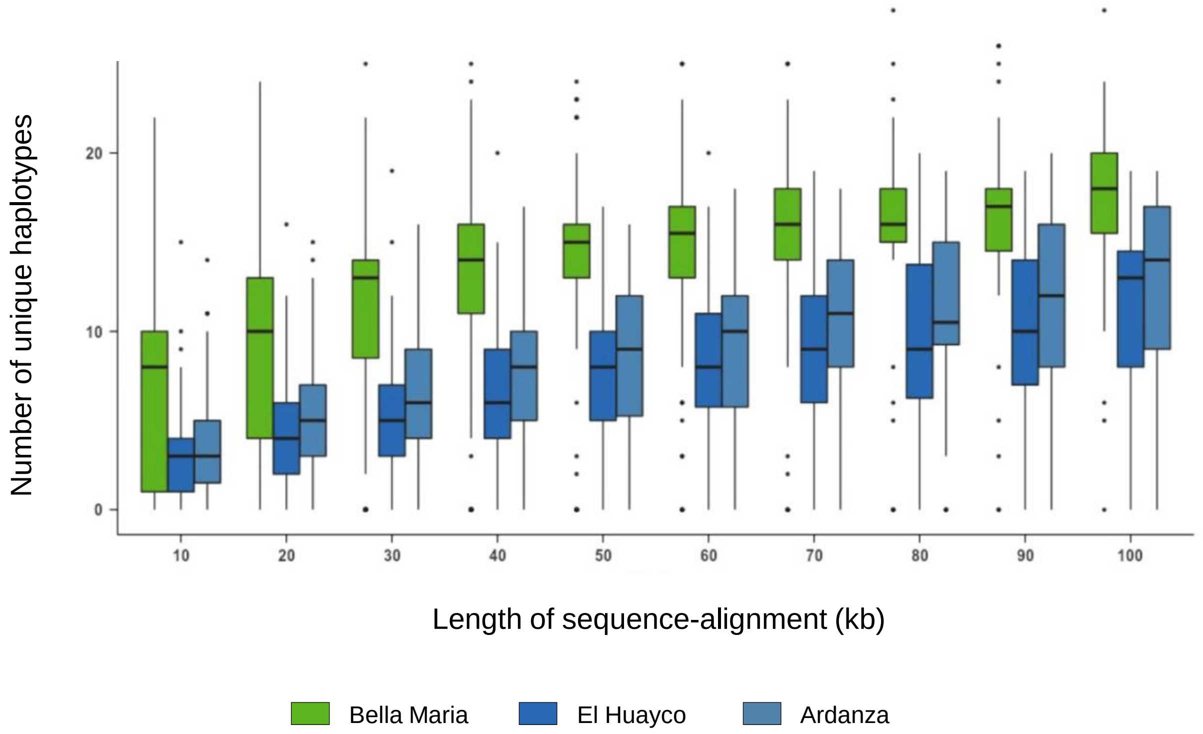

Supplementary Figure 5. Rates of haplotype differentiation relative to sequence length in *T. cruzi* I groups. Boxplots show median and interquartile range for the number of distinct haplotypes found in phased alignment (n = 70,306 SNPs) at window sizes between 0 and 100 kb for Bella Maria, El Huayco and Ardanza groups.

a

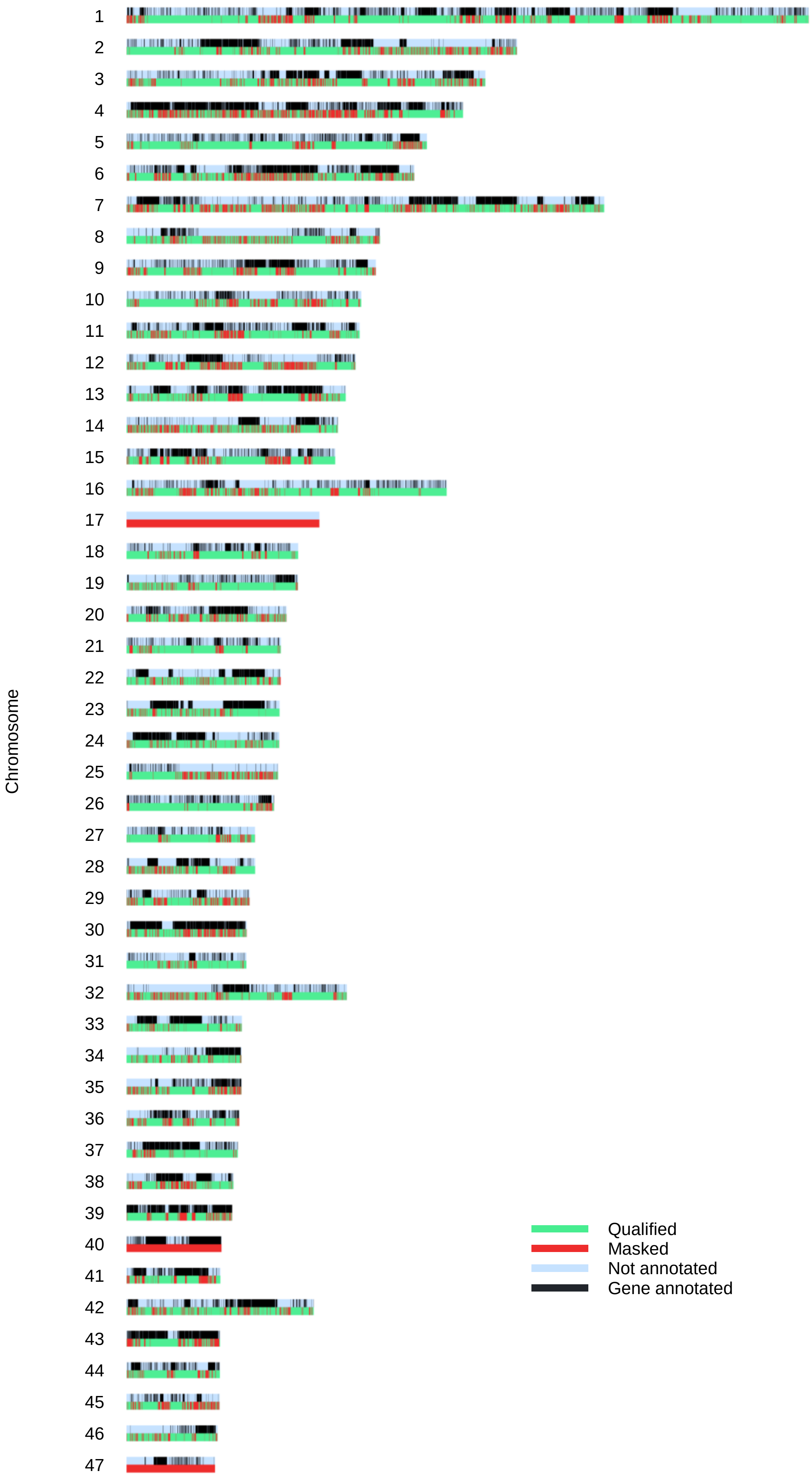

Chromosome
1
2
3
4
5
6
7
8
9
10
11
12
13
14
15
16
17
18
19
20
21
22
23
24
25
26
27
28
29
30
31
32
33
34
35
36
37
38
39
40
41
42
43
44
45
46
47
Qualified
Masked
Not annotated
Gene annotated

b

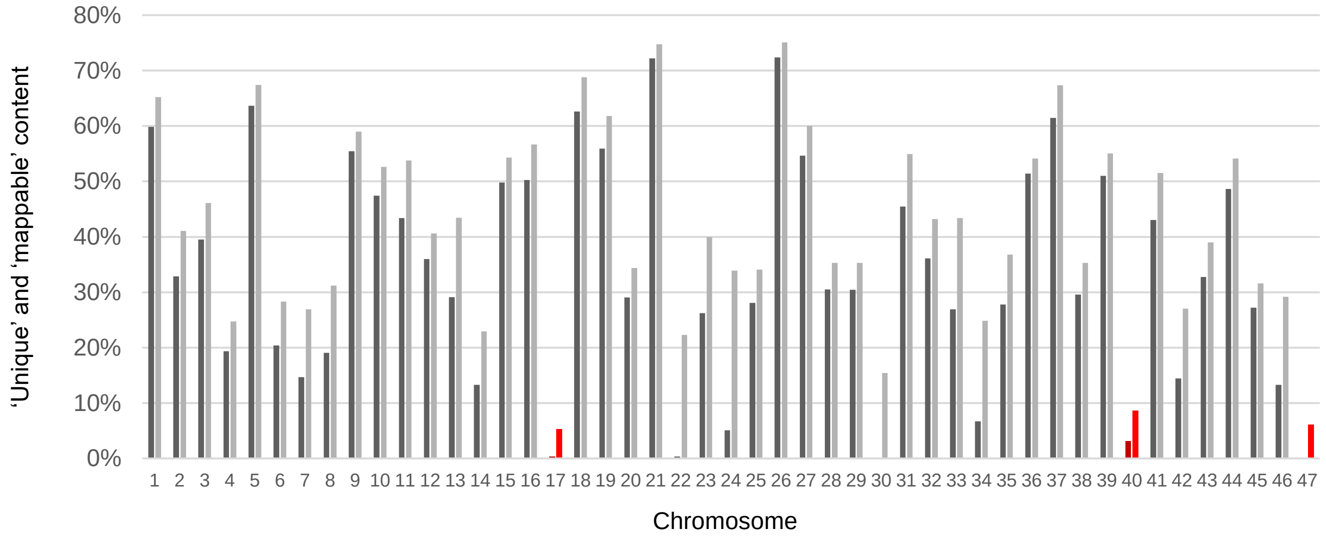

c

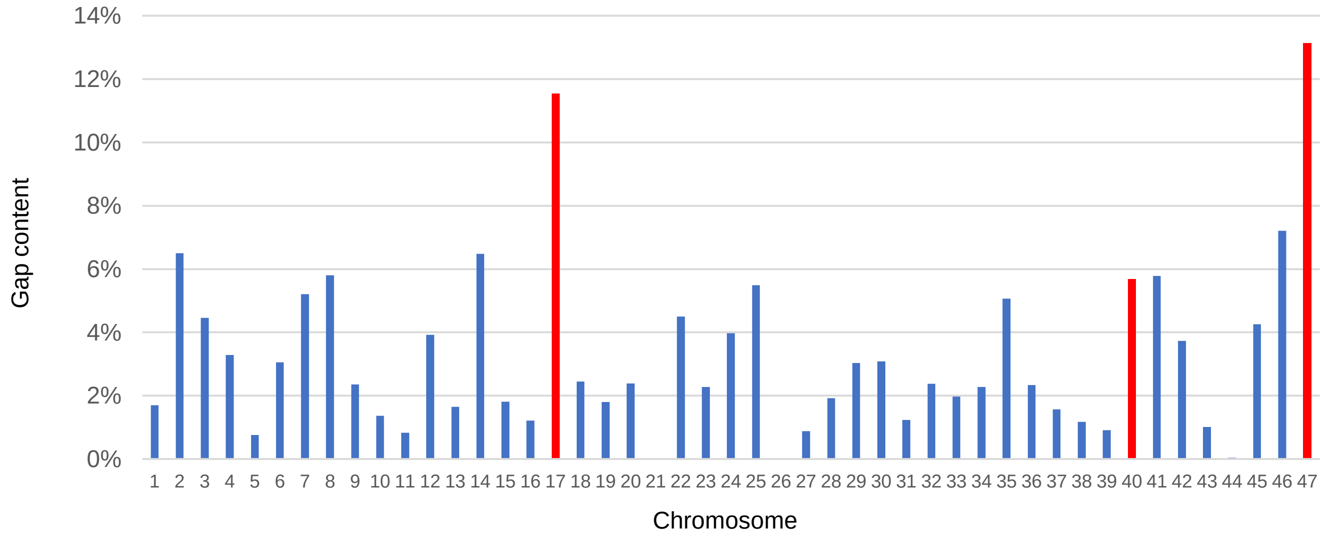

Supplementary Figure 6. TcI-Sylvio reference evaluation and masking. **a** Masks applied to the TcI-Sylvio reference genome based primarily on virtual mappability[4]. Final masking (red) disqualified a total of 24 Mb (including entire chromosomes 17, 40 and 47) of 42 Mb from polymorphism analysis. Annotated genes are marked in black. **b-c** Proportions of mappable[4], unique (determined by self-blasting) and gap content on TcI-Sylvio reference chromosomes are indicated in light grey, dark grey and blue, respectively. Red bars distinguish chromosomes excluded from analysis based on these metrics.

a

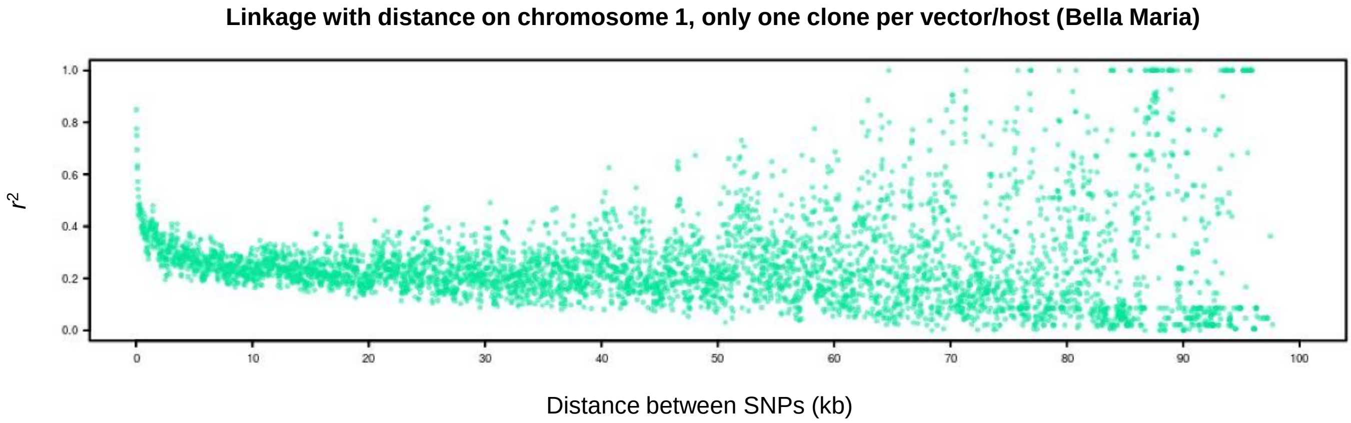

b

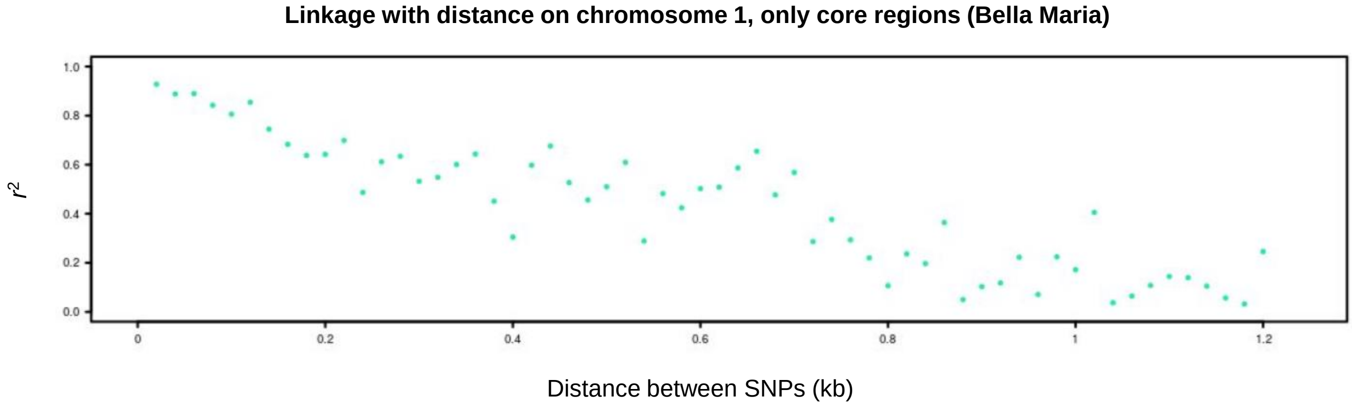

Supplementary Figure 7. Linkage decay in *T. cruzi* I clones from Bella Maria, after sample subsetting. Linkage decay on chromosome 1 remains when analysis is restricted to **a** one random clone per host/vector (*n* = 4,670 SNP sites) or **b** core sequence regions, defined as areas of synteny among TcI-Sylvio, *T. b. brucei* and *L. major* reference genomes. Latter reduction in sample size to 1,178 sites limits analysis to short map distance classes (0 - 1.2 kb). Presentation is otherwise analogous to that in Fig. 3a.

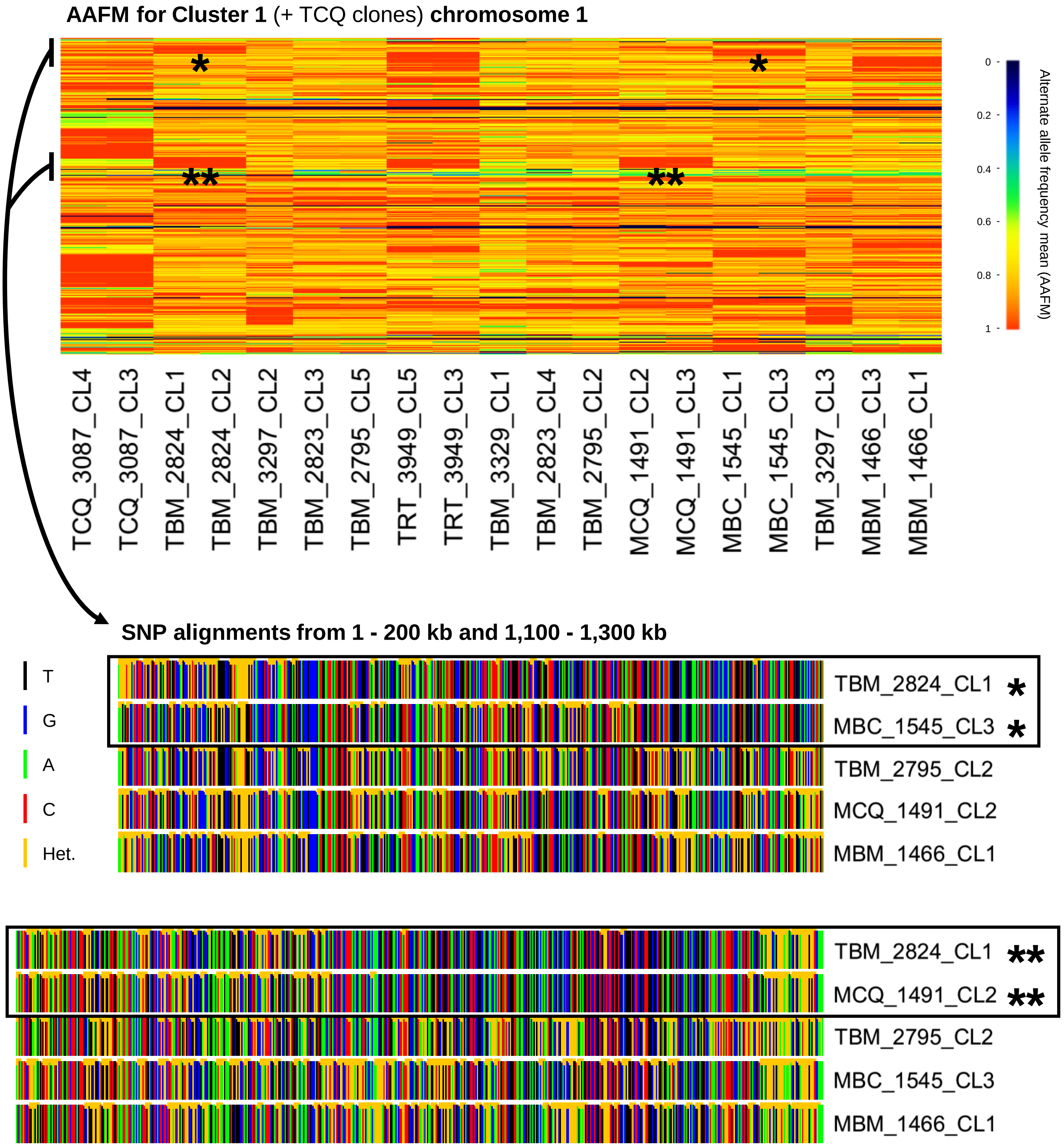

Supplementary Figure 8. Patchy homozygosity and SNP-sharing suggests recombination among *T. cruzi* I clones. In the top plot, each column represents the first chromosome of one clone. Rows within each column represent consecutive 5 kb sequence bins. Alternate allele frequency means (AAFM) determine the color of each bin – blue (0) through green (0.5) to red (1). Long tracts of high AAFM (i.e., large red patches) expose abrupt segmental increases in sequence similarity between different pairs of clones, as exemplified in the SNP concatenations below. Homozygous SNPs are colored according to base identity – black (T), blue (G), green (A) and red (C). Heterozygous SNPs are colored yellow. Single-asterisked AAFM patches reflect high sequence similarity between TBM_2824_CL1 and MBC_1545_CL3 near the start of the chromosome 1. Double-asterisked patches at ca. 1,200 kb reflect a sudden shift in pairwise similarity. Here SNPs in TBM_2824_CL1 and MCQ_1491_CL2 begin to align.

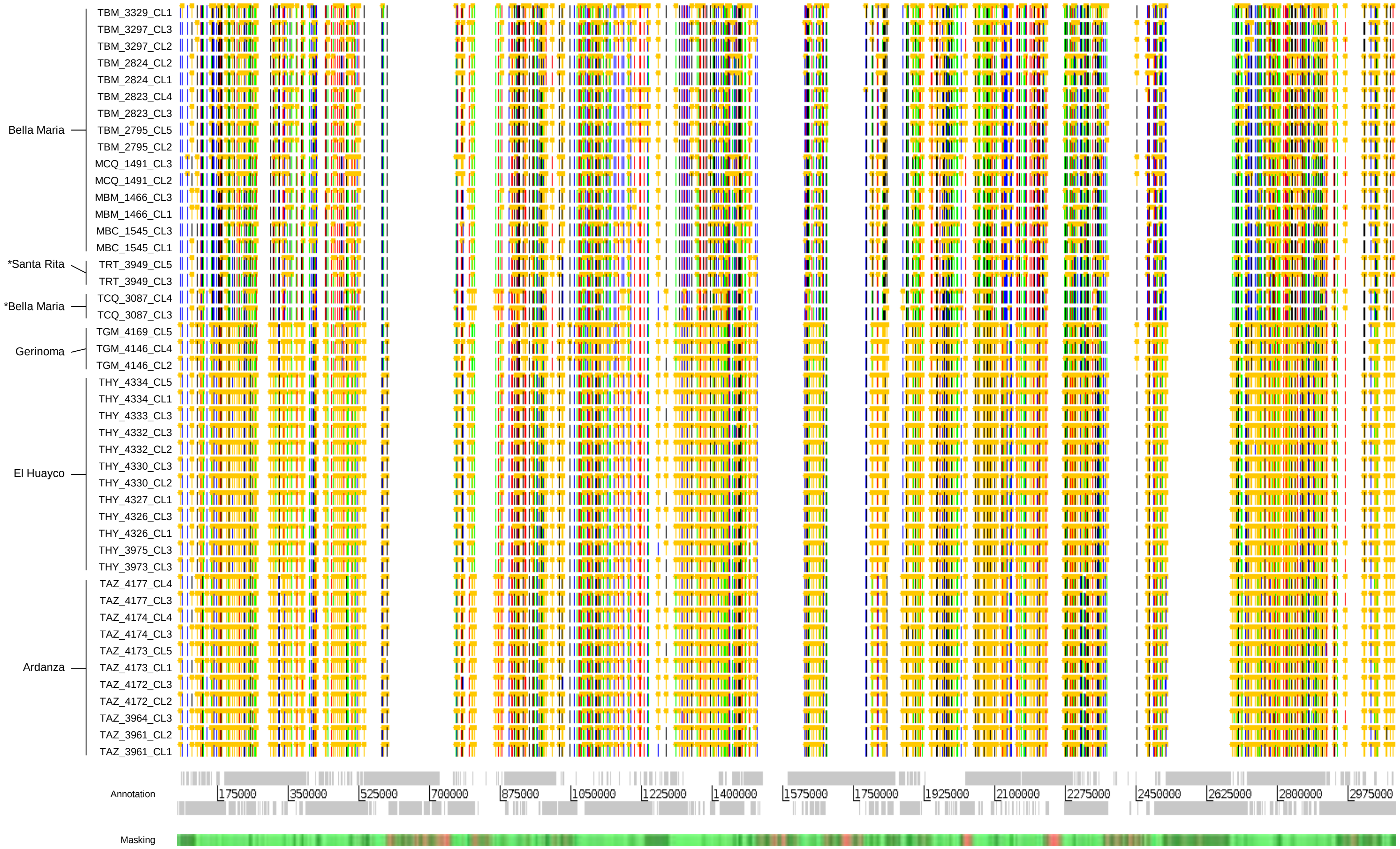

Supplementary Figure 9. SNP alignment across chromosome 1 for all *T. cruzi* I clones. Homozygous SNPs are colored according to base identity – black (T), blue (G), green (A) and red (C). Heterozygous SNPs are colored yellow. Colors overlap where SNP density is high. Only sites without any missing genotypes are shown. White spaces in grey bars below the alignment represent coding regions on forward (top) and reverse (bottom) strands. The third bar indicates masked sequence regions in red, unmasked regions in green.

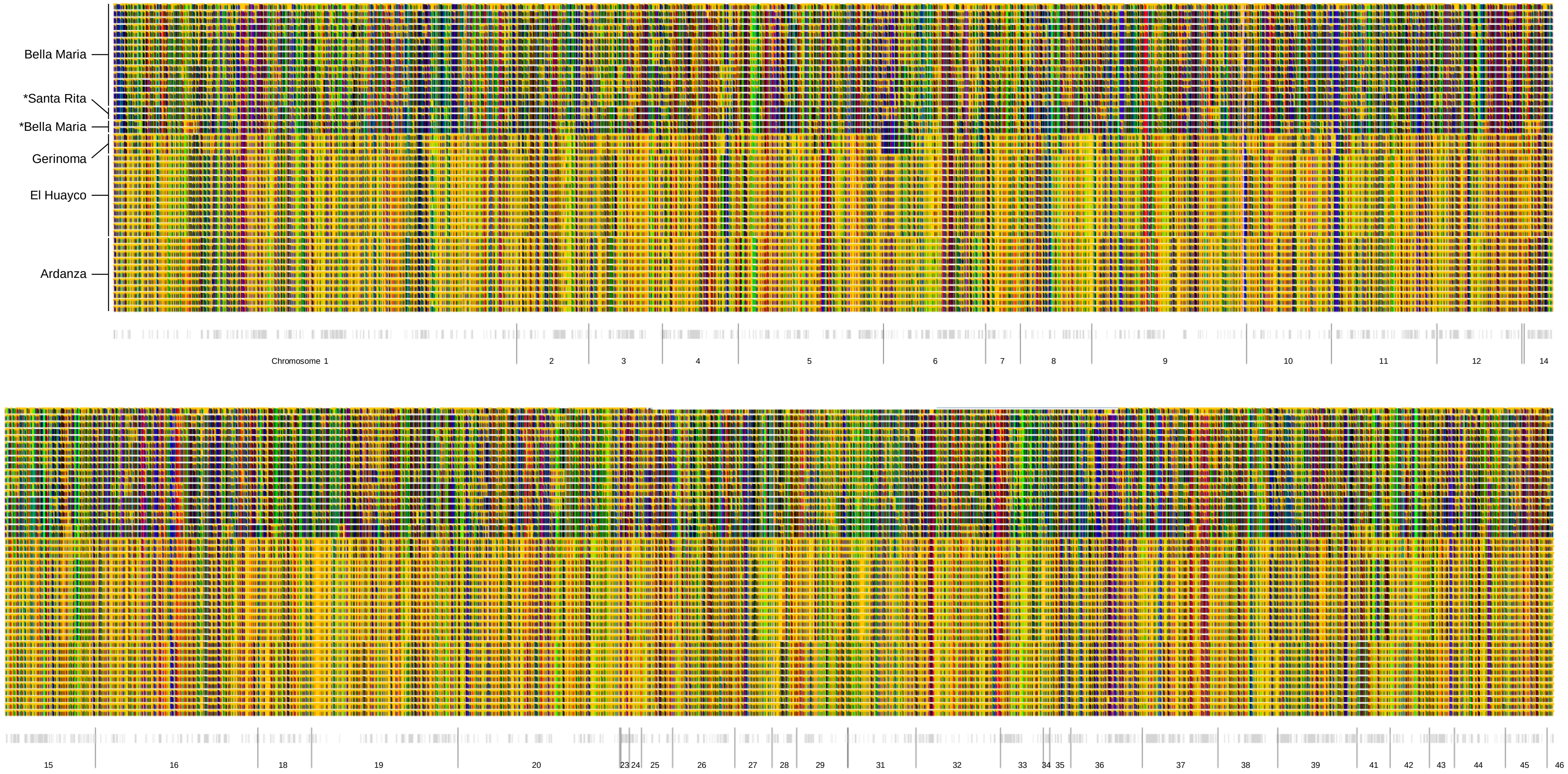

Supplementary Figure 10. Genome-wide SNP alignment for all *T. cruzi* I clones. Homozygous SNPs are colored according to base identity – black (T), blue (G), green (A) and red (C). Heterozygous SNPs are colored yellow. Only variable sites without any missing genotypes are shown. Grey bars below the alignment represent SNPs in coding regions. Asterisks denote outlier samples from Santa Rita (TRT_3949 clones) and Bella Maria (TCQ_3087 clones). Occasional patches of shared homozygosity (e.g., see chromosome 36) in Cluster 2 were not associated to coding vs. non-coding sequence annotation (χ^2 = 0.089, df = 1, p-value = 0.764). Sample order matches that in Supplementary Fig. 9.

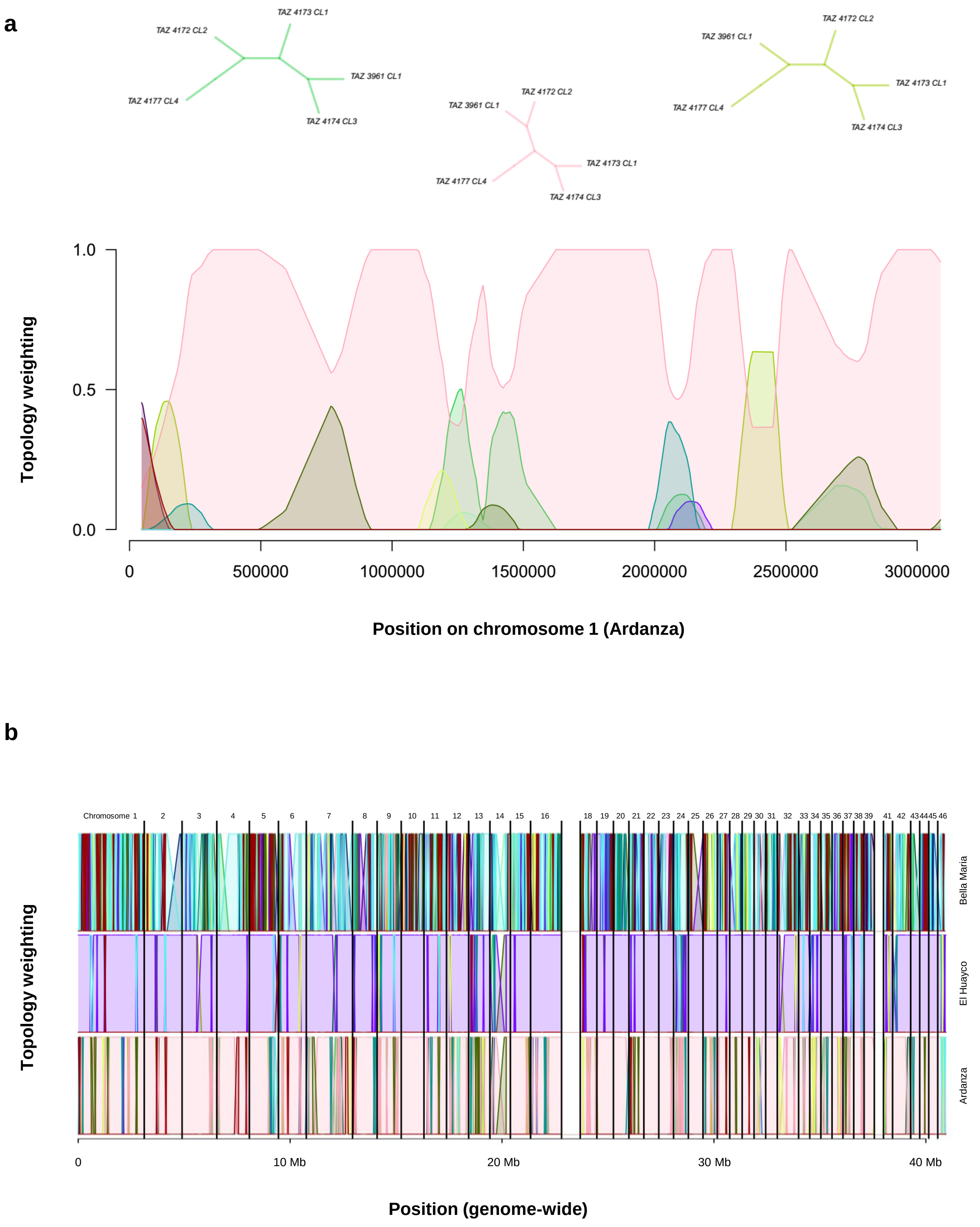

Supplementary Figure 11. Intra-chromosomal phylogenetic relationships among *T. cruzi* I clones. **a** Theoretically, fifteen neighbor-joining (NJ) topologies can be drawn to describe relationships among five samples within a larger phylogenetic tree. When NJ trees are constructed in 50 kb sliding-window analysis (step size = 10 kb), a single topology dominates across chromosome 1 for a five-sample subset from Ardanza. Similar is true for El Huayco (see Fig. 4d). Topology weightings (the relative abundances of the different five-sample topologies after iterative sampling of sub-trees[5]) are plotted (with loess smoothing; span = 0.125) for each window across the chromosome. **b** Mosaic (Bella Maria) vs. stable (Cluster 2) genealogies occur as such genome-wide. Colors represent different tree topologies. Poorly mapping chromosomes 17, 40 and 47 are excluded from analysis).

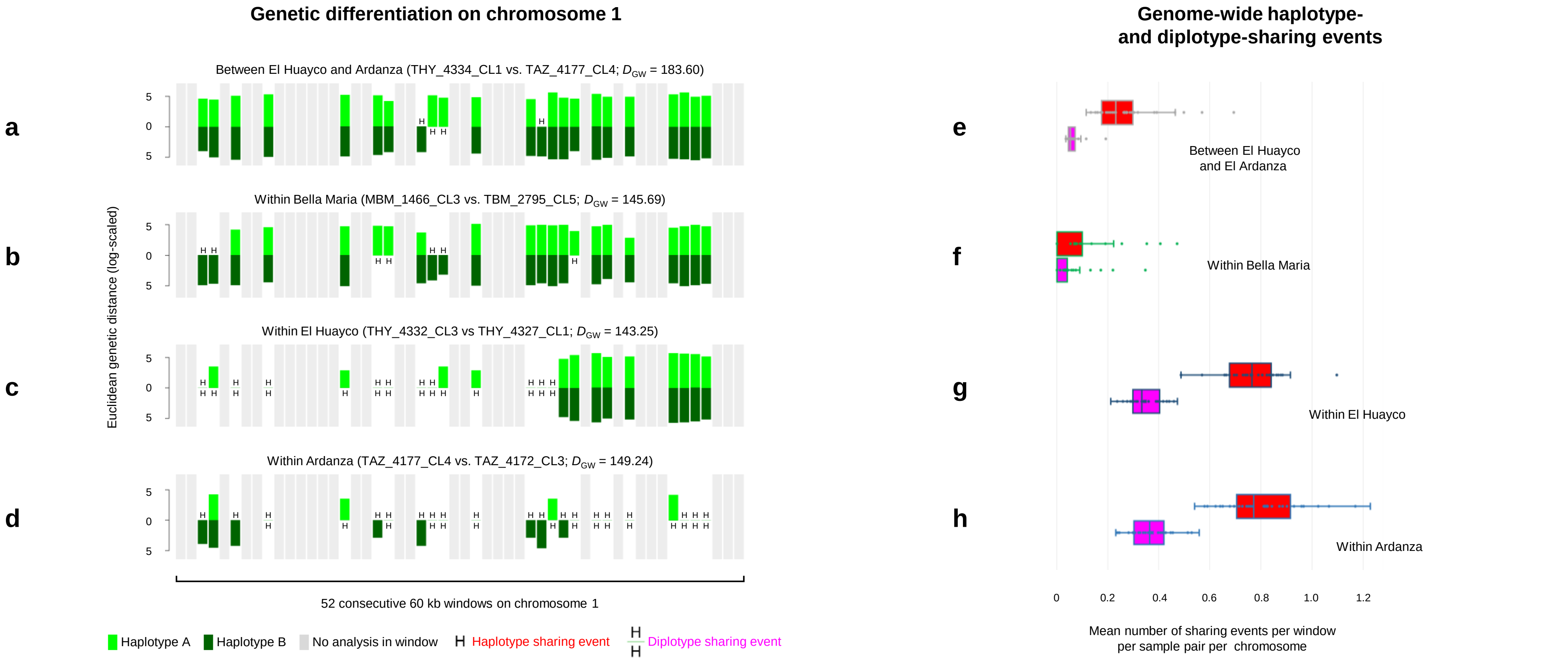

Supplementary Figure 12. Pairwise haplotype and diplotype sharing within and between *T. cruzi* I groups. In plots **a-d**, light green bars indicate genetic distances for pairs of samples in consecutive 60 kb sequence windows along phased haplotype A on chromosome 1 (996 SNP sites). Opposite bars (dark green) quantify distances for haplotype B. Windows of low and/or masked polymorphism (< 20 SNP sites per 60 kb) are shown in grey. These windows are excluded from analysis. **a** compares sample THY 4334 CL1 (El Huayco) to sample TAZ 4177 CL4 (Ardanza) and exemplifies between-group haplotype sharing (marked by the letter H) observed in Cluster 2. Several windows present matching 60 kb haplotypes, i.e., zero differentiation on haplotype A or B. Light or dark green bars therefore do not appear in these windows. **b** shows similar results from a pairwise comparison representative of haplotype differentiation within the Bella Maria group. Pairwise haplotype differentiation within El Huayco (**c**) and within Ardanza (**d**) is different. Shared haplotypes are much more abundant and many windows also present diplotype sharing (marked by two H's), i.e., identical SNP calls on both homologous haplotype segments in both *T. cruzi* clones. Plots **e-h** demonstrate how haplotype (red) and diplotype sharing (pink) events depicted in windowed bar plots for chromosome 1 also occur frequently on other chromosomes and in all possible pairwise comparisons within El Huayco (n = 66 pairwise comparisons) and Ardanza (n = 55). They occur less frequently within the Bella Maria group (n = 105). Each point represents the mean number of sharing events per window per sample pair for one of 37 chromosomes (7,299 sites). Chromosomes 13, 17, 22, 23, 24, 30, 34, 40, 46 and 47 are excluded from analysis due to low polymorphism and/or heavy masking. Vertical bars in boxes and at box edges mark medians and interquartile ranges. D_{GW} is the genome-wide Euclidean genetic distance.

a

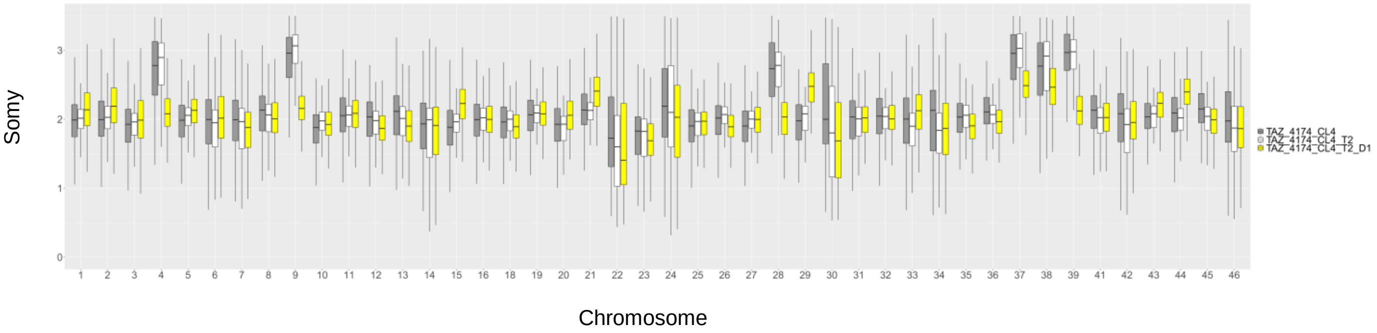

b

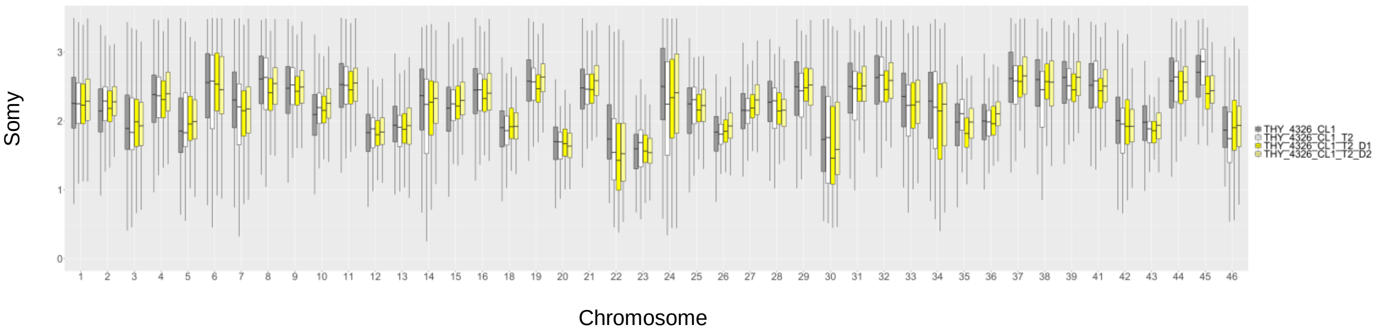

c

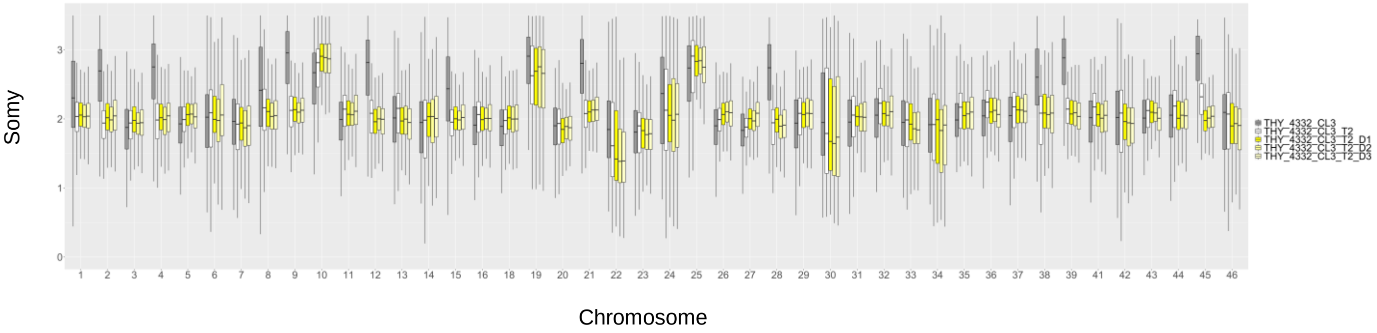

Supplementary Figure 13. Temporal and sub-clonal somy variation for selected *T. cruzi* I clones. **a** Following first sequencing and sample cryopreservation, TAZ_4174_CL4 was thawed, re-expanded in Liver Infusion Tryptose (LIT) medium (no additional passages) and sequenced for a second time. One subclone obtained from the re-cultured sample was also sequenced. Boxplots show median and interquartile range of site-wise somy estimates ($2 \cdot m$ / p30 of M_m) for each chromosome (see *Methods*). While the 'parent' clone karyotype appeared unchanged at time of second sequencing (T2), results for subclone T2_D1 suggest sub-clonal chromosomal copy number variation (e.g., see white vs. yellow boxplots for chromosomes 4 and 39). **b** THY_4326_CL1 was also re-sequenced but showed no evidence of somy differences between subclones (n = 2) or over time. The sample was passaged three times post-cryopreservation. **c** THY_4332_CL3 appeared to have reduced somy levels between first and second sequencing (four passages), but no sub-clonal variation was observed (n = 3).

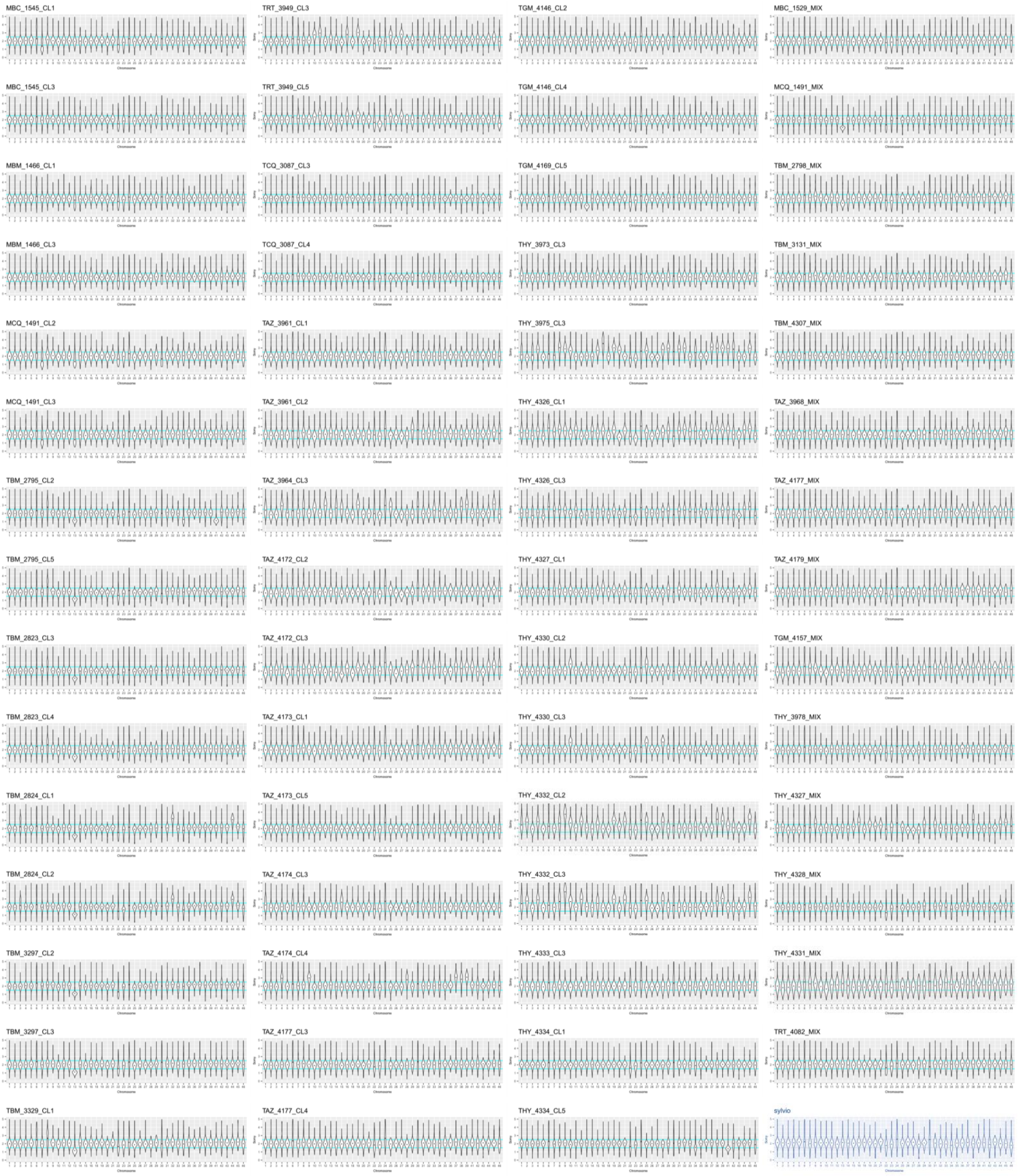

Supplementary Figure 14. Somy estimates for cloned and non-cloned *T. cruzi* samples. Somy variation among samples was inferred from sequence read-depth kernel density distributions. Dots within violin plots indicate the median of site-wise somy estimates ($2 \cdot m$ / p30 of M_m) for each chromosome (see *Methods*). Results for the TcI-Sylvio reference validate the procedure. Results for non-cloned, low-diversity infections (e.g., MCQ_1491_MIX and THY_4327_MIX) suggest that aneuploidies in clones are not consequences of plate-cloning procedures in the lab.

a

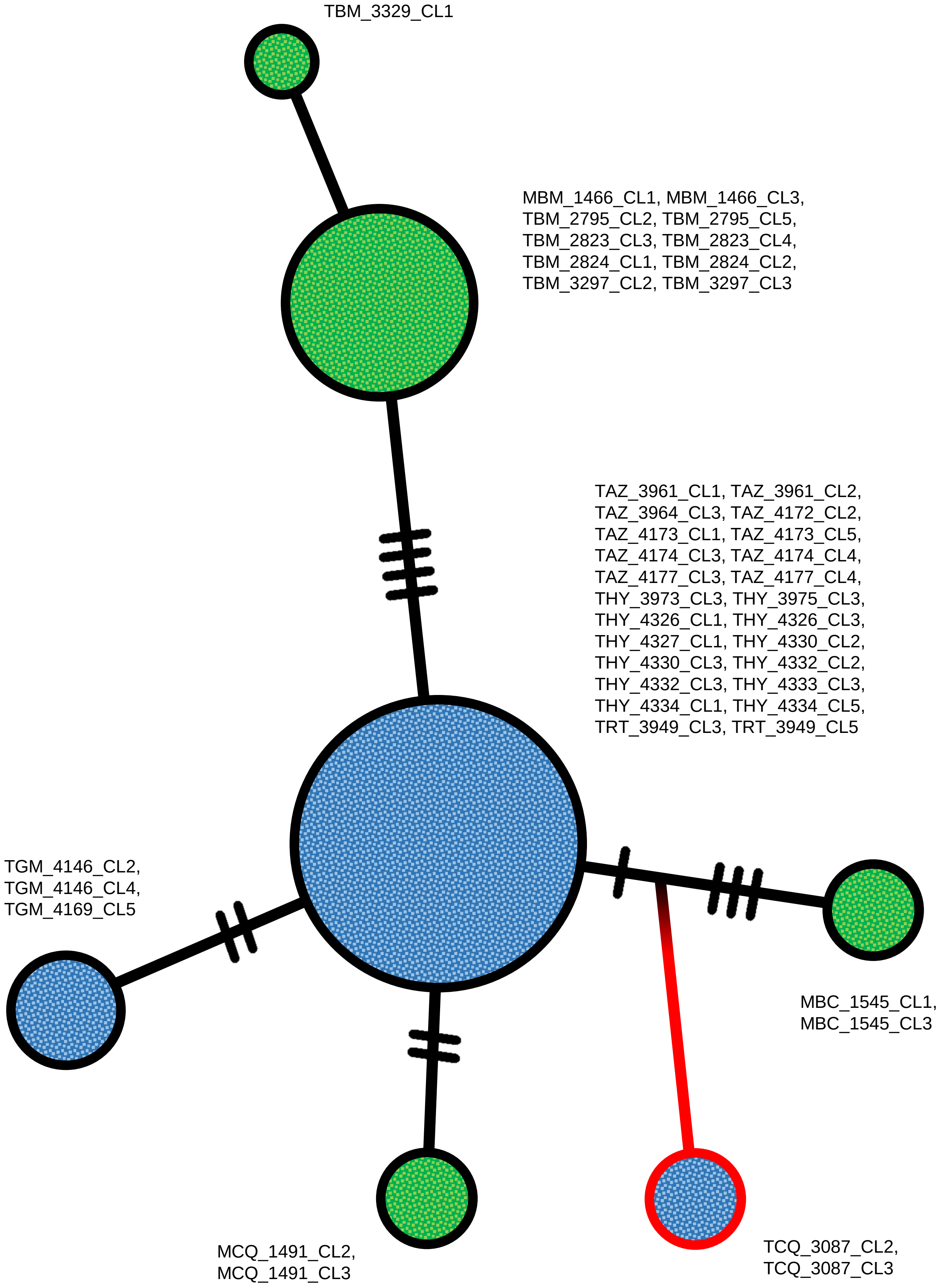
TBM_3329_CL1
MBM_1466_CL1, MBM_1466_CL3, TBM_2795_CL2, TBM_2795_CL5, TBM_2823_CL3, TBM_2823_CL4, TBM_2824_CL1, TBM_2824_CL2, TBM_3297_CL2, TBM_3297_CL3
TAZ_3961_CL1, TAZ_3961_CL2, TAZ_3964_CL3, TAZ_4172_CL2, TAZ_4173_CL1, TAZ_4173_CL5, TAZ_4174_CL3, TAZ_4174_CL4, TAZ_4177_CL3, TAZ_4177_CL4, THY_3973_CL3, THY_3975_CL3, THY_4326_CL1, THY_4326_CL3, THY_4327_CL1, THY_4330_CL2, THY_4330_CL3, THY_4332_CL2, THY_4332_CL3, THY_4333_CL3, THY_4334_CL1, THY_4334_CL5, TRT_3949_CL3, TRT_3949_CL5
TGM_4146_CL2, TGM_4146_CL4, TGM_4169_CL5
MBC_1545_CL1, MBC_1545_CL3
MCQ_1491_CL2, MCQ_1491_CL3
TCQ_3087_CL2, TCQ_3087_CL3

b

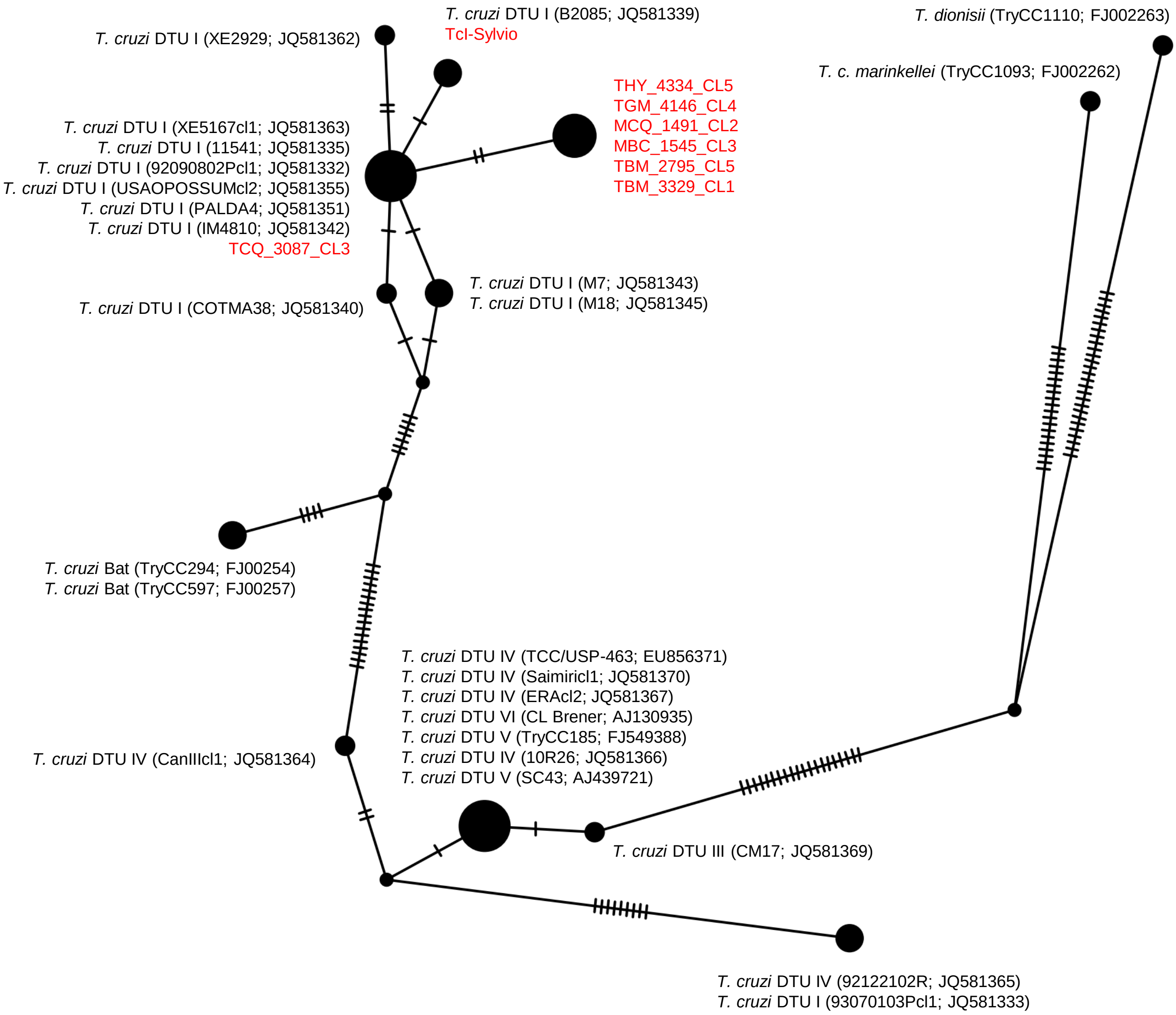

Supplementary Figure 15. Mitochondrial phylogenies for *T. cruzi* I clones. **a** Maxicircle sequence variation among all samples except TAZ_4172_CL3 (missing information at 44% SNP sites) represented as a TCS network[6]. Black tick marks between nodes indicate the number of mutations between genotypes. Node sizes correspond to the number of samples represented by the particular maxicircle variant. Green nodes contain members of Cluster 1, as defined in nuclear phylogenetic analysis (Fig. 1). Blue nodes contain members of Cluster 2, but also TRT_3949 clones. TCQ_3087 clones appear divergent, with 668 diagnostic SNP differences relative to other clones of Cluster 2. **b** TCS network from *cytochrome b* alignment (617 bp), for which sequences from all 7 *T. cruzi* sublineages and various congeners are available for comparison. These sequences are detailed in Messenger *et al.* 2012[7] and Marcili *et al.* 2009[8]. Tick marks indicate number of mutations between genotypes. Samples from this study (one representative per maxicircle variant) are shown in red.

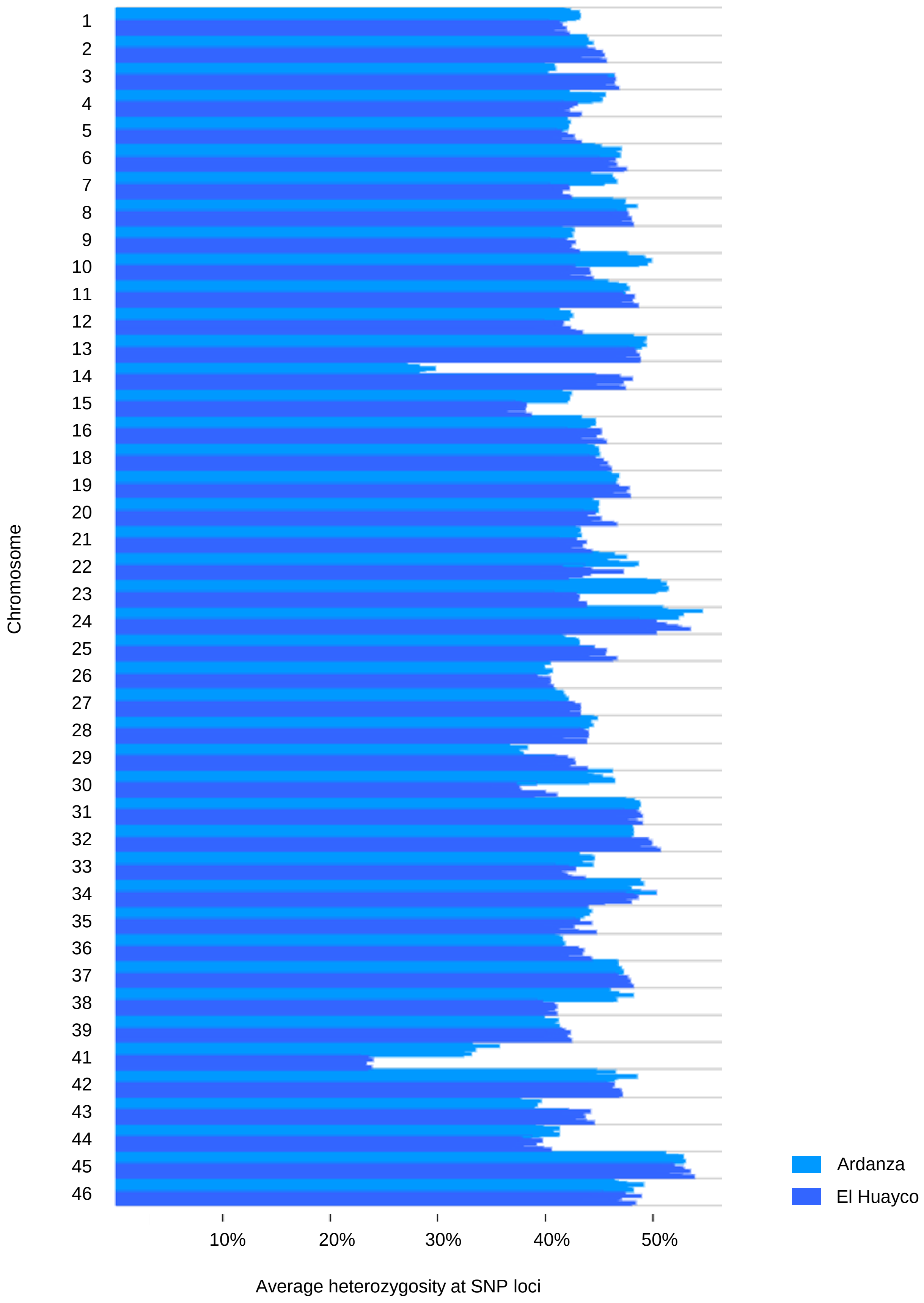

Supplementary Figure 16. Heterozygosity per chromosome in *T. cruzi* I clones from El Huayco and Ardanza. Average heterozygosity values fall between 40 and 50% for most chromosomes. Only chromosomes 14 (in Ardanza clones) and 41 (in both Ardanza and El Huayco clones) show substantial increases in homozygosity.

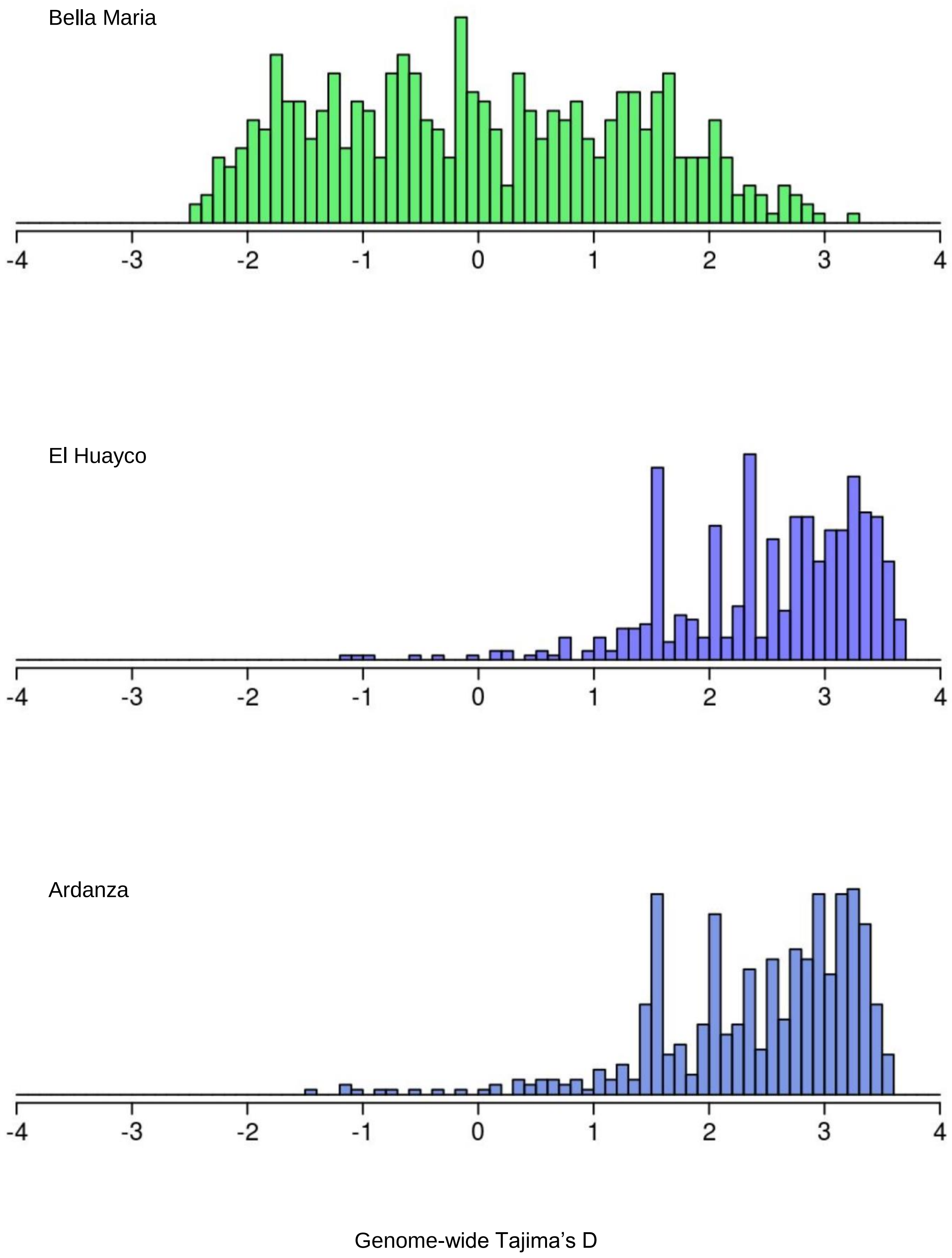

Supplementary Figure 17. SNP variation relative to neutral expectations in T*.* *cruzi* I groups. Histograms plot variation in Tajima's D values over 50 kb sequence bins in genomes from Bella Maria (96,691 SNPs), El Huayco (80,052 SNPs) and Ardanza (78,325 SNPs). Empty bins (i.e., windows lacking polymorphism within the group) do not enter analysis.

Supplementary Table 1 Host/vector sampling sites and *T. cruzi* I genomic sequencing coverage

ID	Longitude (°)	Latitude (°)	Altitude (m)	Region	Ecotype	Host	Year	NRD	MRD
MBC_1545_CL1	-79.6039	-4.2134	1143	Bella Maria	sylvatic	*Artibeus fraterculus*	2012	23.3	75.4
MBC_1545_CL3	-79.6039	-4.2134	1143	Bella Maria	sylvatic	*Artibeus fraterculus*	2012	24.8	114.0
MBM_1466_CL1	-79.6166	-4.2185	1376	Bella Maria	sylvatic	*Rhipidomys leucodactylus*	2012	22.4	92.2
MBM_1466_CL3	-79.6166	-4.2185	1376	Bella Maria	sylvatic	*Rhipidomys leucodactylus*	2012	20.5	110.0
MCQ_1491_CL2	-79.5995	-4.2241	1272	Bella Maria	sylvatic	*Simosciurus nebouxii*	2012	16.4	167.4
MCQ_1491_CL3	-79.5995	-4.2241	1272	Bella Maria	sylvatic	*Simosciurus nebouxii*	2012	18.8	112.6
TAZ_3961_CL1	-79.5969	-4.2878	1311	Ardanza	domestic	*Panstrongylus rufotuberculatus*	2015	15.1	34.3
TAZ_3961_CL2	-79.5969	-4.2878	1311	Ardanza	domestic	*Panstrongylus rufotuberculatus*	2015	15.1	47.1
TAZ_3964_CL3	-79.5969	-4.2878	1311	Ardanza	domestic	*Panstrongylus rufotuberculatus*	2015	13.6	50.5
TAZ_4172_CL2	-79.5969	-4.2878	1311	Ardanza	domestic	*Panstrongylus rufotuberculatus*	2015	29.8	26.8
TAZ_4172_CL3	-79.5969	-4.2878	1311	Ardanza	domestic	*Panstrongylus rufotuberculatus*	2015	24.7	6.0
TAZ_4173_CL1	-79.5969	-4.2878	1311	Ardanza	domestic	*Panstrongylus rufotuberculatus*	2015	14.0	23.5
TAZ_4173_CL5	-79.5969	-4.2878	1311	Ardanza	domestic	*Panstrongylus rufotuberculatus*	2015	23.0	57.7
TAZ_4174_CL3	-79.5969	-4.2878	1311	Ardanza	domestic	*Panstrongylus rufotuberculatus*	2015	16.0	59.5
TAZ_4174_CL4	-79.5969	-4.2878	1311	Ardanza	domestic	*Panstrongylus rufotuberculatus*	2015	22.8	116.3
TAZ_4177_CL3	-79.5969	-4.2878	1311	Ardanza	domestic	*Panstrongylus rufotuberculatus*	2015	21.4	47.0
TAZ_4177_CL4	-79.5969	-4.2878	1311	Ardanza	domestic	*Panstrongylus rufotuberculatus*	2015	16.1	65.9
TBM_2795_CL2	-79.6063	-4.2115	1132	Bella Maria	domestic	*Panstrongylus chinai*	2011	25.9	149.2
TBM_2795_CL5	-79.6063	-4.2115	1132	Bella Maria	domestic	*Panstrongylus chinai*	2011	55.1	406.7
TBM_2823_CL3	-79.6063	-4.2115	1132	Bella Maria	domestic	*Panstrongylus chinai*	2011	53.5	185.3
TBM_2823_CL4	-79.6063	-4.2115	1132	Bella Maria	domestic	*Panstrongylus chinai*	2011	17.3	71.6
TBM_2824_CL1	-79.6063	-4.2115	1132	Bella Maria	domestic	*Panstrongylus chinai*	2011	50.9	296.7
TBM_2824_CL2	-79.6063	-4.2115	1132	Bella Maria	domestic	*Panstrongylus chinai*	2011	53.8	180.0
TBM_3297_CL2	-79.5985	-4.2285	1265	Bella Maria	sylvatic	*Rhodnius ecuadoriensis*	2013	64.0	474.7
TBM_3297_CL3	-79.5985	-4.2285	1265	Bella Maria	sylvatic	*Rhodnius ecuadoriensis*	2013	21.7	154.2
TBM_3329_CL1	-79.6170	-4.2085	1379	Bella Maria	sylvatic	*Rhodnius ecuadoriensis*	2013	18.5	61.3
TCQ_3087_CL3	-79.5972	-4.2258	1203	Bella Maria	sylvatic	*Rhodnius ecuadoriensis*	2012	54.6	580.8
TCQ_3087_CL4	-79.5972	-4.2258	1203	Bella Maria	sylvatic	*Rhodnius ecuadoriensis*	2012	45.7	301.4
TGM_4146_CL2	-79.4635	-4.0952	1801	Gerinoma	peri-domestic	*Triatoma carrioni*	2015	12.7	53.3
TGM_4146_CL4	-79.4635	-4.0952	1801	Gerinoma	peri-domestic	*Triatoma carrioni*	2015	19.5	86.5
TGM_4169_CL5	-79.4635	-4.0952	1801	Gerinoma	peri-domestic	*Triatoma carrioni*	2015	19.5	67.4
THY_3973_CL3	-79.3188	-4.0906	1375	El Huayco	sylvatic	*Rhodnius ecuadoriensis*	2015	13.6	55.1
THY_3975_CL3	-79.3188	-4.0906	1375	El Huayco	sylvatic	*Rhodnius ecuadoriensis*	2015	25.2	129.9
THY_4326_CL1	-79.3188	-4.0906	1375	El Huayco	sylvatic	*Rhodnius ecuadoriensis*	2015	16.9	34.3
THY_4326_CL3	-79.3188	-4.0906	1375	El Huayco	sylvatic	*Rhodnius ecuadoriensis*	2015	36.9	82.3
THY_4327_CL1	-79.3188	-4.0906	1375	El Huayco	sylvatic	*Rhodnius ecuadoriensis*	2015	16.7	82.3
THY_4330_CL2	-79.3188	-4.0906	1375	El Huayco	sylvatic	*Rhodnius ecuadoriensis*	2015	16.3	70.7
THY_4330_CL3	-79.3188	-4.0906	1375	El Huayco	sylvatic	*Rhodnius ecuadoriensis*	2015	25.8	125.5
THY_4332_CL2	-79.3188	-4.0906	1375	El Huayco	sylvatic	*Rhodnius ecuadoriensis*	2015	20.8	28.0
THY_4332_CL3	-79.3188	-4.0906	1375	El Huayco	sylvatic	*Rhodnius ecuadoriensis*	2015	22.8	12.4
THY_4333_CL3	-79.3188	-4.0906	1375	El Huayco	sylvatic	*Rhodnius ecuadoriensis*	2015	15.1	68.1
THY_4334_CL1	-79.3188	-4.0906	1375	El Huayco	sylvatic	*Rhodnius ecuadoriensis*	2015	27.2	118.7
THY_4334_CL5	-79.3188	-4.0906	1375	El Huayco	sylvatic	*Rhodnius ecuadoriensis*	2015	36.2	255.8
TRT_3949_CL3	-79.3475	-4.1125	1278	Santa Rita	domestic	*Panstrongylus chinai*	2015	20.2	88.1
TRT_3949_CL5	-79.3475	-4.1125	1278	Santa Rita	domestic	*Panstrongylus chinai*	2015	18.9	68.8

Abbreviations: NRD (average nuclear read-depth); MRD (average maxicircle read-depth).

Supplementary Table 2 Examples of long tracts of homozygosity found in *T. cruzi* I genomes

ID	Chromosome	Start pos. (bp)	End pos. (bp)	No. of variants	No. of mismatches	Region	Cluster
MBC_1545_CL1	1	2527616	2735662	377	1	Bella Maria	1
MBC_1545_CL1	3	688123	992700	193	0	Bella Maria	1
MBC_1545_CL1	3	688123	992700	204	0	Bella Maria	1
MBC_1545_CL1	4	1078068	1217217	98	3	Bella Maria	1
MBC_1545_CL1	5	244489	367798	226	0	Bella Maria	1
MBC_1545_CL1	5	642558	754816	540	1	Bella Maria	1
MBC_1545_CL1	7	330951	911251	282	2	Bella Maria	1
MBC_1545_CL1	7	934166	1174673	793	0	Bella Maria	1
MBC_1545_CL1	10	74632	241049	910	1	Bella Maria	1
MBC_1545_CL1	10	553233	691425	89	1	Bella Maria	1
MBC_1545_CL1	10	752929	1034308	766	3	Bella Maria	1
MBC_1545_CL1	15	106823	318213	1304	3	Bella Maria	1
MBC_1545_CL1	15	510886	782403	925	1	Bella Maria	1
MBC_1545_CL1	15	784193	934142	659	0	Bella Maria	1
MBC_1545_CL1	16	206821	502967	544	1	Bella Maria	1
MBC_1545_CL1	19	235735	342459	289	5	Bella Maria	1
MBC_1545_CL1	19	625331	769267	826	1	Bella Maria	1
MBC_1545_CL1	31	323674	456229	766	7	Bella Maria	1
MBC_1545_CL1	35	213894	501036	803	0	Bella Maria	1
MBC_1545_CL1	36	99405	228968	448	0	Bella Maria	1
MBC_1545_CL1	36	99405	228968	894	0	Bella Maria	1
MBC_1545_CL1	41	135171	239973	513	0	Bella Maria	1
MBC_1545_CL1	41	135171	239973	1023	0	Bella Maria	1
MBC_1545_CL1	42	394695	618542	513	5	Bella Maria	1
MCQ_1491_CL2	1	590428	761231	10	0	Bella Maria	1
MCQ_1491_CL2	1	1494729	1618252	64	3	Bella Maria	1
MCQ_1491_CL2	1	1494729	1618252	68	0	Bella Maria	1
MCQ_1491_CL2	1	1494729	1618252	69	0	Bella Maria	1
MCQ_1491_CL2	1	2527893	2702894	123	4	Bella Maria	1
MCQ_1491_CL2	3	1190725	1409940	625	5	Bella Maria	1
MCQ_1491_CL2	4	1097976	1222344	27	0	Bella Maria	1
MCQ_1491_CL2	7	934166	1196685	262	0	Bella Maria	1
MCQ_1491_CL2	10	351472	525227	182	0	Bella Maria	1
MCQ_1491_CL2	10	750071	1059591	799	2	Bella Maria	1
MCQ_1491_CL2	12	615126	831143	32	0	Bella Maria	1
MCQ_1491_CL2	12	615126	831143	62	0	Bella Maria	1
MCQ_1491_CL2	13	380831	571325	672	2	Bella Maria	1
MCQ_1491_CL2	13	380831	571325	694	0	Bella Maria	1
MCQ_1491_CL2	13	590920	891408	285	1	Bella Maria	1
MCQ_1491_CL2	13	590920	891408	290	0	Bella Maria	1
MCQ_1491_CL2	14	768946	873091	352	0	Bella Maria	1
MCQ_1491_CL2	16	923925	1076491	615	0	Bella Maria	1
MCQ_1491_CL2	25	693	273362	1039	4	Bella Maria	1
MCQ_1491_CL2	27	46321	205232	627	2	Bella Maria	1
MCQ_1491_CL2	28	405150	507611	139	2	Bella Maria	1
TAZ_3961_CL2	7	925153	1113043	177	3	Ardanza	2
THY_4330_CL3	35	21625	153853	22	0	El Huayco	2

This table summarizes long tracts of homozygosity found in MBC_1545_CL1 and MCQ_1491_CL2, two clones that typify homozygosity patterns in the Bella Maria group. Clones from El Huayco and Ardanza except THY_4330_CL3 and TAZ_3961_CL2 (one single occurrence each) entirely lack these tracts.

Supplementary Table 3 Recalculation of population genetic descriptive metrics using only one random *T. cruzi* I clone per vector/host

Group (*n*)	PS	Median π	Median θ	PS at MAF > 0.05	PRS (vs. BM / EH / AR)	SS	PS in HW Equilibrium	HS	Fixed HS
Bella Maria (8)	95,313	0.13	0.001	59 %	0 / 41,270 / 41,063	22,344	90,461	55,571	2,848
El Huayco (8)	76,889	0.53	0.001	71 %	22,846 / 0 / 17,681	6,855	44,911	56,016	45,792
Ardanza (6)	75,709	0.55	0.001	71 %	21,459 / 16,501 / 0	9,844	72,968	55,638	47,761

We reduced the dataset to identify biases related to multiple- vs. single-clone sampling per infection. While overall inference is similar, single-clone sampling can raise estimates of nucleotide diversity and rates of type II error in Hardy-Weinberg equilibrium null hypothesis testing (see power analysis in Supplementary Fig. 4). Abbreviations: PS (polymorphic sites); π (nucleotide diversity, per site); θ (Watterson estimator, per site); MAF (within-group minor allele frequency); PRS (private sites); SS (singleton sites); HW (Hardy-Weinberg); HS (heterozygous sites).

Supplementary Table 4 Re-sequencing of clones and subclones for additional ploidy analyses

ID	Type	No. of passages in LIT	RL	NRD
TAZ_4174_CL4_T2	Clone	0	2 x 75	135
THY_4326_CL1_T2	Clone	3	2 x 75	131
THY_4332_CL3_T2	Clone	4	2 x 75	180
TAZ_4174_CL4_T2_D1	Subclone	0	2 x 150	31
THY_4326_CL1_T2_D1	Subclone	0	2 x 150	49
THY_4326_CL1_T2_D2	Subclone	0	2 x 150	26
THY_4332_CL3_T2_D1	Subclone	0	2 x 150	44
THY_4332_CL3_T2_D2	Subclone	0	2 x 150	59
THY_4332_CL3_T2_D3	Subclone	0	2 x 150	41

Having entered cryopreservation (-150 °C) immediately after the first epimastigote DNA extraction (Dec. 2016), three clones were re-expanded into liquid culture and further subcloned by limiting dilution starting Dec. 2018. These clones and subclones underwent ≤ 4 passages in Liver Infusion Tryptose (LIT) medium prior to epimastigote DNA extraction in Mar. 2019. Abbreviations: RL (read-length); NRD (average nuclear read-depth).

Supplementary References

1. Ward, J. H. Hierarchical grouping to optimize an objective function. *J. Am. Stat. Assoc.* **58**, 236 (1963).

2. Danecek, P. *et al.* The variant call format and VCFtools. *Bioinformatics* **27**, 2156–2158 (2011).

3. Ewing, A. D. *et al.* Combining tumor genome simulation with crowdsourcing to benchmark somatic single-nucleotide-variant detection. *Nat. Methods* **12**, 623–630 (2015).

4. Derrien, T. *et al.* Fast computation and applications of genome mappability. *PLoS One* **7**, (2012).

5. Martin, S. H. & Van Belleghem, S. M. Exploring evolutionary relationships across the genome using topology weighting. *Genetics* **206**, 429–438 (2017).

6. Clement, M., Posada, D. & Crandall, K. A. TCS: a computer program to estimate gene genealogies. *Mol. Ecol.* **9**, 1657–1659 (2000).

7. Messenger, L. A. *et al.* Multiple mitochondrial introgression events and heteroplasmy in *Trypanosoma cruzi* revealed by maxicircle MLST and next generation sequencing. *PLoS Negl. Trop. Dis.* **6**, e1584 (2012).

8. Marcili, A. *et al.* A new genotype of *Trypanosoma cruzi* associated with bats evidenced by phylogenetic analyses using *SSU* rDNA, *cytochrome b* and *Histone H2B* genes and genotyping based on *ITS1* rDNA. *Parasitology* **136**, 641–655 (2009).
