# Supplementary material for: Meiotic sex in Chagas disease parasite Trypanosoma cruzi
Source: Nat Commun. 2019 Sep 3;10:3972. doi: 10.1038/s41467-019-11771-z (PMC6722143; doi:10.1038/s41467-019-11771-z)
Supplement: Supplementary file 3 — Reporting Summary [file 41467_2019_11771_MOESM3_ESM.pdf]

nature research

Corresponding author(s): Martin Llewellyn

Reporting Summary

Nature Research wishes to improve the reproducibility of the work that we publish. This form provides structure for consistency and transparency in reporting. For further information on Nature Research policies, see Authors & Referees and the Editorial Policy Checklist.

Statistical parameters

When statistical analyses are reported, confirm that the following items are present in the relevant location (e.g. figure legend, table legend, main text, or Methods section).

n/a	Confirmed	
☐	☒	The exact sample size (*n*) for each experimental group/condition, given as a discrete number and unit of measurement
☐	☒	An indication of whether measurements were taken from distinct samples or whether the same sample was measured repeatedly
☒	☐	The statistical test(s) used AND whether they are one- or two-sided<br>*Only common tests should be described solely by name; describe more complex techniques in the Methods section.*
☒	☐	A description of all covariates tested
☒	☐	A description of any assumptions or corrections, such as tests of normality and adjustment for multiple comparisons
☐	☒	A full description of the statistics including central tendency (e.g. means) or other basic estimates (e.g. regression coefficient) AND variation (e.g. standard deviation) or associated estimates of uncertainty (e.g. confidence intervals)
☒	☐	For null hypothesis testing, the test statistic (e.g. *F*, *t*, *r*) with confidence intervals, effect sizes, degrees of freedom and *P* value noted<br>*Give P values as exact values whenever suitable.*
☐	☒	For Bayesian analysis, information on the choice of priors and Markov chain Monte Carlo settings
☒	☐	For hierarchical and complex designs, identification of the appropriate level for tests and full reporting of outcomes
☒	☐	Estimates of effect sizes (e.g. Cohen's *d*, Pearson's *r*), indicating how they were calculated
☐	☒	Clearly defined error bars<br>*State explicitly what error bars represent (e.g. SD, SE, CI)*

Our web collection on statistics for biologists may be useful.

Software and code

Policy information about availability of computer code

Data collection	n/a
Data analysis	Smalt v.0.7.4 (hash-based sequence read alignment); BWA-mem v0.7.3 (suffix tree-based sequence read alignment); Picard v1.85 (high-throughput sequence data handling); Genome Analysis Toolkit (GATK) v3.7.0 (genetic variant discovery and genotyping); Genomic Multi-tool v1.376 (indexing and querying genomic data files); BEAGLE v4.1 (haplotype phasing by use of localized haplotype clustering); SplitsTree v4 (phylogenetic network analysis); fineSTRUCTURE v2.0.4 (Bayesian inference of population structure from dense sequencing data); ape v5.173 (phylogenetic analyses in R); hierfstat v0.04-2274 (F-statistics calculation in R); adegenet 2.1.1 (multivariate analysis of genetic markers in R); PLINK v1.90b3w (genome analysis association toolset); VCFtools v0.1.13 (variant-call-format data handling); LDhat (population genetic analysis of recombination); BAMSurgeon v1.0.0 (synthetic mutation introduction to sequence alignment maps); fastsimcoal2 v5.2.8 (coalescent simulation of genomic diversity); PopART v1.7 (population analysis with reticulate trees), Twisst/PhyML v3.1 (phylogenetic topology weighting), Artemis v.16.0.0 (sequence visualization)

For manuscripts utilizing custom algorithms or software that are central to the research but not yet described in published literature, software must be made available to editors/reviewers upon request. We strongly encourage code deposition in a community repository (e.g. GitHub). See the Nature Research guidelines for submitting code & software for further information.

Data

Policy information about availability of data

All manuscripts must include a data availability statement. This statement should provide the following information, where applicable:

- Accession codes, unique identifiers, or web links for publicly available datasets
- A list of figures that have associated raw data
- A description of any restrictions on data availability

Sequence data that support the findings of this study are available at Sequence Read Archive (SRA) BioProject PRJNA552612 [https://www.ncbi.nlm.nih.gov/sra/PRJNA552612]. All other relevant data are available from the corresponding author on reasonable request.

Field-specific reporting

Please select the best fit for your research. If you are not sure, read the appropriate sections before making your selection.

☒ Life sciences ☐ Behavioural & social sciences ☐ Ecological, evolutionary & environmental sciences

For a reference copy of the document with all sections, see nature.com/authors/policies/ReportingSummary-flat.pdf

Life sciences study design

All studies must disclose on these points even when the disclosure is negative.

Sample size	Genomic data from 59 samples (45 clones and 14 non-cloned strains) were analyzed in this study. Sequencing coverage averaged 27x. Three clones were resequenced to assess genomic plasticity. Multiple subclones of these three samples were also generated and sequenced to assess heterogeneity among individual cells, providing unprecedented levels of replication and resolution in the study of Trypanosoma cruzi population genetics.
Data exclusions	Genomic sequencing failed for 24 samples with low DNA quantity/quality. These did not proceed into any genomic analysis.
Replication	Genomic data from 59 samples (45 clones and 14 non-cloned strains) were analyzed in this study. Sequencing coverage averaged 27x. Three clones were resequenced to assess genomic plasticity. Multiple subclones of these three samples were also generated and sequenced to assess heterogeneity among individual cells. Several analyses were also performed separately for independent chromosomes (n = 44) or re-assessed in specific sequence subcategories (e.g., orthologous regions). Various statistical re-sampling methods (e.g., bootstrapping, comparisons of different spatial sampling configurations, etc.) were also applied as indicated in the manuscript.
Randomization	This case study does not involve experimental groups or require randomized treatment or measurement.
Blinding	Operator bias is not relevant to the population genetic analyses performed in this study.

Reporting for specific materials, systems and methods

Materials & experimental systems

n/a	Involved in the study
☒	☐ Unique biological materials
☒	☐ Antibodies
☐	☒ Eukaryotic cell lines
☒	☐ Palaeontology
☒	☐ Animals and other organisms
☒	☐ Human research participants

Methods

n/a	Involved in the study
☒	☐ ChIP-seq
☒	☐ Flow cytometry
☒	☐ MRI-based neuroimaging

Eukaryotic cell lines

Policy information about cell lines

Cell line source(s)	Illumina sequence reads for Trypanosoma cruzi TcI X10/1 Sylvio were provided by Carlos Talavera-López, SciLifeLab, Sweden.
Authentication	Sequence polymorphism comparison to PacBio reference assembly.

Mycoplasma contamination	n/a
Commonly misidentified lines (See ICLAC register)	n/a
